# Genetic diversity of pneumococcal surface protein A in invasive pneumococcal isolates from Korean children, 1991-2016

**DOI:** 10.1371/journal.pone.0183968

**Published:** 2017-11-13

**Authors:** Ki Wook Yun, Eun Hwa Choi, Hoan Jong Lee

**Affiliations:** 1 Department of Pediatrics, Seoul National University College of Medicine, Seoul, Korea; 2 Department of Pediatrics, Seoul National University Children’s Hospital, Seoul, Korea; Universidad Nacional de la Plata, ARGENTINA

## Abstract

Pneumococcal surface protein A (PspA) is an important virulence factor of pneumococci and has been investigated as a primary component of a capsular serotype-independent pneumococcal vaccine. Thus, we sought to determine the genetic diversity of PspA to explore its potential as a vaccine candidate. Among the 190 invasive pneumococcal isolates collected from Korean children between 1991 and 2016, two (1.1%) isolates were found to have no pspA by multiple polymerase chain reactions. The full length *pspA* genes from 185 pneumococcal isolates were sequenced. The length of *pspA* varied, ranging from 1,719 to 2,301 base pairs with 55.7–100% nucleotide identity. Based on the sequences of the clade-defining regions, 68.7% and 49.7% were in PspA family 2 and clade 3/family 2, respectively. PspA clade types were correlated with genotypes using multilocus sequence typing and divided into several subclades based on diversity analysis of the N-terminal α-helical regions, which showed nucleotide sequence identities of 45.7–100% and amino acid sequence identities of 23.1–100%. Putative antigenicity plots were also diverse among individual clades and subclades. The differences in antigenicity patterns were concentrated within the N-terminal 120 amino acids. In conclusion, the N-terminal α-helical domain, which is known to be the major immunogenic portion of PspA, is genetically variable and should be further evaluated for antigenic differences and cross-reactivity between various PspA types from pneumococcal isolates.

## Introduction

*Streptococcus pneumoniae* is a major cause of community-acquired infectious diseases such as otitis media, pneumonia, bacteremia, and meningitis in children and adults [1]. Pneumococcus consists of more than 94 different serotypes that are determined by the polysaccharide capsule, which is the target of pneumococcal conjugate vaccines (PCVs) currently in use. The introduction of a 7-valent PCV (PCV7, Prevenar^®^, Wyeth Pharmaceuticals) in the early 2000s followed by the extended-valency PCVs, namely, a 10-valent PCV (PCV10, Synflorix^®^, GlaxoSmithKline) and a 13-valent PCV (PCV13, Prevenar13^®^, Pfizer), in the late 2000s has led to a reduction of invasive pneumococcal diseases (IPD) and carriage due to the serotypes included in the PCVs; however, medically relevant replacement by non-vaccine serotypes (NVTs) has been reported [2–4].

The low serotype coverage and the high cost of PCVs have hampered their implementation, especially in developing countries; therefore, the development of a serotype-independent vaccine has been suggested. Many pneumococcal proteins have been investigated as vaccine candidates, such as pneumolysin (Ply), pneumococcal histidine triad protein D (PhtD), and pneumococcal surface protein A (PspA) [5,6].

PspA is an important virulence factor that interferes with complement deposition on the pneumococcal surface and is detected on almost all pneumococci. PspA has a complex mosaic structure comprising five domains, which includes a signal peptide, an α-helical charged domain that includes a clade-defining region, a proline-rich domain, a choline-binding domain consisting of ten 20-amino-acid repeats, and a C-terminal 17-amino-acid tail. Classification by family and clade is based on the amino acid identity of the clade-defining region. The α-helical domain of PspA is exposed on the surface and is therefore able to interact with the human host [7].

PspA is known to be highly immunogenic and induces cross-reactive immunity among different genotypes [8,9]. However, the genetic diversity of PspA should be fully explored prior to its implementation as a vaccine candidate. Although the molecular epidemiology of PspA has been analyzed in some countries, the numbers of pneumococcal isolates and collection periods remain limited [10–12]. The aim of this study was to characterize the genetic diversity of PspA, one of the most promising antigens for a protein vaccine, in a large collection of invasive pneumococcal isolates obtained from children between 1991 and 2016.

## Materials and methods

This study was approved by the Institutional Review Board of Seoul National University Children’s Hospital (SNUCH; IRB registration number 1306-071-527). The Ethics Committee waived informed consent because this study included only the bacterial information without any information regarding the patients from whom the bacteria were obtained.

### Strains

A total of 190 invasive pneumococcal isolates were obtained from children <18 years of age at the SNUCH between 1991 and 2016. An ‘invasive isolate’ was defined as an isolate obtained from a normally sterile body fluid, such as blood, cerebrospinal fluid, pleural fluid, ascites, or joint fluid. Each isolate was identified using standard microbiological techniques, including observations of colony morphology, hemolysis patterns, and optochin susceptibility tests. Additionally, all of the study isolates were confirmed as pneumococci by polymerase chain reaction (PCR) and sequencing of pneumolysin as described in a previous study [6]. Serotypes were determined using the Quellung reaction and multiplex PCR followed by sequencing of the capsular genes [13]. For conventional clade typing, six additional PspA sequences (BG9739, Rx1, EF3296, BG7561, ATCC6303, and BG6380) in a previous study [7] were retrieved from the GenBank database and were used as the reference sequences for PspA clades 1 to 6, respectively.

### Detection and sequencing of PspA

Extraction and purification of DNA from pneumococcal colonies were performed using a QIAamp kit (QIAGEN GmbH, Hilden, Germany) according to the manufacturer’s protocol. To obtain the entire sequence of *pspA*, multiple sets of new primers in addition to primers from a previous study [7] were used (Table 1). PCR was performed in 20 μL volumes, with each reaction mixture containing the following: 2.0 μL of 10× Tris-HCl buffer (100 mM, pH 8.3, Mg^2+^ free), 1.6 μL of 2.5 mM dNTPs, 1.4 μL of MgCl_2_, 0.2 μL of 5.0 U/μL Taq DNA polymerase (Takara Bio Inc., Shiga, Japan), and 4.0 μM of each primer. Thermal cycling was performed in a PTC-200 Peltier Thermal Cycler DNA engine (MJ Research, Watertown, MA) under the following conditions: 95°C for 5 min followed by 35 amplification cycles of 95°C for 30 sec, 58°C for 30 sec, and 72°C for 90 sec, with a final extension at 72°C for 10 min.

**Table 1:** Oligonucleotide primers used for PCR and sequencing in this study.

Primer name	Sequence (5’ → 3’)	Reference
LSM2	GCGCGTCGACGGCTTAAACCCATTCACCATTGG	[7]
LSM12	CCGGATCCAGCGTCGCTATCTTAGGGGCTGGTT	
LSM13	GCAAGCTTATGATATAGAAATTTGTAAC	
SKH2	CCACATACCGTTTTCTTGTTTCCAGCC	
PspA-1F	AAAGATTGTCCGCAGGCTTA	This study
PspA-1R	AAAATGTCAAATGTTCTTAACATGC	
PspA-2-1F	AACCAGAGAAGCCAGCTGAA	
PspA-2-2F	AAGAAACTCCAGCTCCAGCA	
PspA-2-3F	AGCTGCTGAAGCTGAGTTGG	
PspA-3F	GACAAATATTTACGGAGGAGGCTA	
PspA-1-1R	AAAATGTCAAATGTTCTTAACATGC	
PspA-2R	TTGAAGGTCGTGTGTGCTTC	
PspA-3R	ATCACATCGTAGCCCTGCTC	

An isolate was designated negative for *pspA* if no gene product was amplified using any of the primers, including LSM12 and SKH2, which are thought to amplify all known *pspA* genes [14]. Sequence analyses of the *pspA* genes were performed for 185 (97.4%) invasive pneumococcal isolates harboring *pspA*. Sequence data from the *pspA* fragments obtained from each strain were assembled and edited using Sequencher (Gene Codes Inc., Ann Arbor, MI). Further editing, alignment, and additional analyses were performed using CLC Main Workbench ver. 6.6.5 software (CLC bio, Aarhus, Denmark). All sequences generated in this study have been deposited in GenBank under accession numbers KY446182 to KY446366.

### PspA clade and family typing

The amino acid sequence was translated from each nucleotide sequence. The clade and family types were determined from the amino acid sequences of the PspA clade-defining region [7]. The sequences of 185 pneumococcal isolates and the reference strains for each clade were grouped based on diversity. Clade type was established when the sequences shared a common branch with the corresponding reference strain on the dendrogram. Clades 1 and 2 were placed into family 1, clades 3–5 to family 2, and clade 6 to family 3 [7]. The prevalence and distribution of the family and clade types from different study periods, serotypes, and genotypes were analyzed. The diversities of the PspA clades by serotype including more than two isolates were estimated using Simpson’s index of diversity D as previously described [15].

### Analysis of genetic diversity of the α-helical domain

The entire N-terminal α-helical domain, including the clade-defining region, was extracted from the full sequence of PspA according to a previous study [7]. To investigate the inter- and intra-clade sequence diversities of the α-helical domain, alignments of amino acid sequences were performed using the pairwise comparison method, and dendrograms were constructed using the maximum likelihood reconstruction method with the WAG substitution model. The percentages of replicate trees in which the associated sequences clustered together in the bootstrap test (1,000 replicates) are reported as the bootstrap values on the main internal nodes of the tree. Additionally, we assigned subclades alphabetically to each clade type based on the dendrogram. A subclade was defined as a group of sequences sharing a common branch divided from the main internal nodes. All of these analyses were performed using CLC Main Workbench ver. 6.6.5 software.

### Putative antigenicity plots

The antigenic patterns of the α-helical domain were predicted using CLC Main Workbench ver. 6.6.5 software for all 185 PspAs. The amino acid sequences of the most recent isolates in each PspA subclade were converted to the corresponding antigenicity plot. This conversion was accomplished by assigning a hydrophobicity value to each amino acid and then calculating a moving average of these values along the peptide chain. The point of the highest local average hydrophobicity was invariably located in or immediately adjacent to an antigenic determinant [16]. Antigenicity values were calculated, and the relevant diagrams were constructed. Antigenicity plots with different amplitudes or numbers of peak hydrophobicity points were defined as having ‘different antigenicity’.

### Multilocus sequence typing

Multilocus sequence typing (MLST) was performed with partial datasets. Invasive pneumococcal isolates obtained from children <5 years of age between 1995 and 2005 were retrieved from a previous study [17]. Additionally, pneumococci serogroups 6 and 19 have been analyzed using MLST via a surveillance program ongoing at SNUCH since 1991 for genetic structures of major invasive pneumococcal serotypes in children.

## Results

### Isolates

A total of 190 invasive pneumococcal isolates were obtained from Korean children at a single center over 26 years. Among them, 78.4% (n = 149) were isolated from blood, 9.5% (n = 18) from cerebrospinal fluid, 5.8% (n = 11) from lung tissue or pleural fluid, 4.7% (n = 9) from bone tissue or joint fluid, and 1.6% (n = 3) from ascites. Twenty-four (12.6%) isolates were collected from 1991–1995, 54 (28.3%) isolates from 1996–2000, 47 (24.6%) isolates from 2001–2005, 40 (20.9%) isolates from 2006–2010, and 26 (13.6%) isolates from 2011–2016. A total of 30 serotypes were identified; the most common serotype was 19A (n = 32, 16.8%), followed by 23F (n = 25, 13.2%), 6B (n = 17, 8.9%), and 14 (n = 16, 8.4%). Eighty-five (44.7%) isolates were PCV7 types (serotypes 4, 6B, 9V, 14, 18C, 19F, and 23F), 52 (27.4%) isolates were PCV13 additional types (serotypes 1, 3, 5, 6A, 7F, and 19A), and the remaining 53 (27.9%) isolates were NVTs.

### PspA full sequence variation

**Fig 1:**
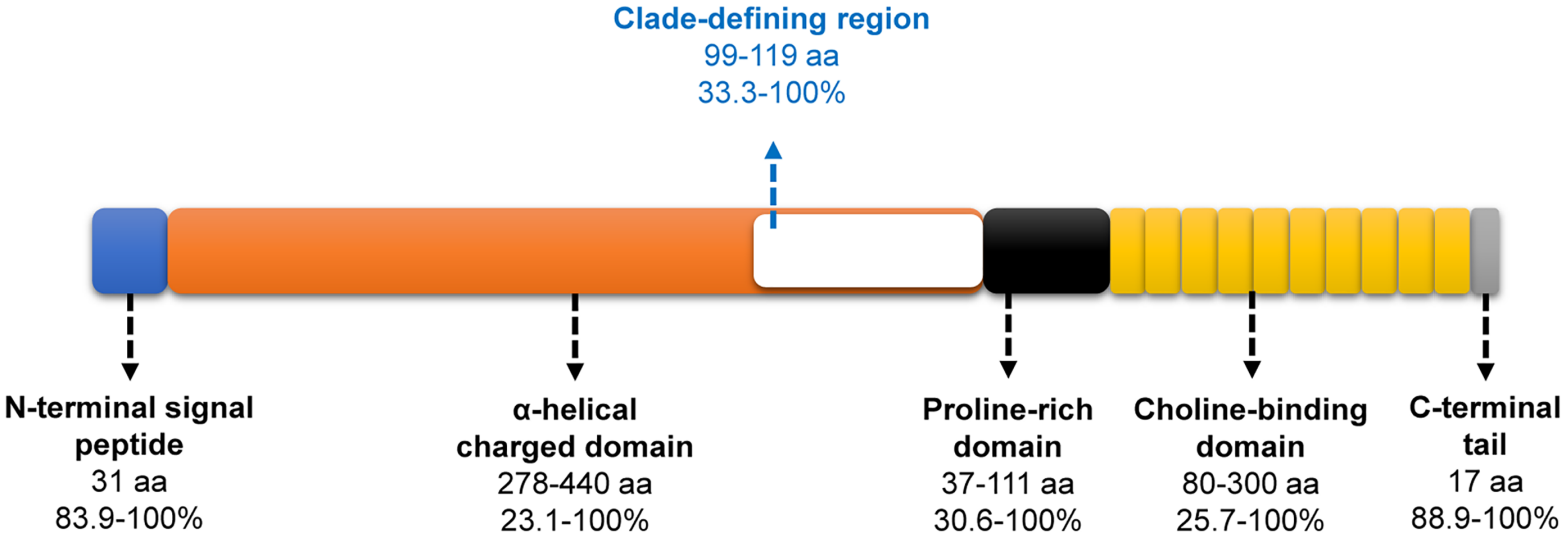
Genomic structure of PspA analyzed in this study. Colored boxes indicate the five domains of PspA, including the clade-defining region (white box). At the end of each arrow extending from the domains, the corresponding name, size variation, and sequence identity of the amino acids are presented. In the choline-binding domain, the small yellow box indicates the 20-amino-acid repeat unit, which was repeated 4–15 times in the genes of the 185 study isolates.

All isolates of *S*. *pneumoniae* except for two (98.9%) contained the *pspA* gene. We amplified and analyzed the complete sequence of 185 *pspA* genes. The nucleotide sequences of the remaining three *pspA* genes were each ambiguous in a portion of the sequence and were thus excluded from the list of completely sequenced *pspA* genes. There were 55 different sizes of *pspA* genes, ranging from 1,719 to 2,301 base pairs (bps). An allele type with 2,175 bp was the most common (n = 36, 19.5%). The entire span of *pspA* showed 44.4–100% nucleotide identity and 31.6–100% amino acid identity. All *pspA* sequences can be divided into five previously known domains (Fig 1). The sizes of the N-terminal signal peptide and C-terminal tail were 31 and 17 amino acids, respectively, for all of the genes. The amino acid sequence identities of the signal peptide (83.9–100%) and tail (88.9–100%) were highest among the five PspA domains.

The amino acid sequences of the α-helical and proline-rich domains were variable with 278–440 and 37–111 amino acids, and with 23.1–100% and 30.6–100% identities, respectively. The well-known 20-amino-acid repeat in the choline-binding domain of PspA was variably repeated 4–15 times (80–300 amino acids) with amino acid sequence identities of 25.7–100%. Ten repeats were the most common (n = 78, 42.2%), followed by 11 (n = 55, 29.7%), 9 (n = 38, 20.5%), 8 (n = 7, 3.8%), 12 (n = 5, 2.7%), 15 (n = 1, 0.5%), and 4 (n = 1, 0.5%) repeats.

### PspA clade and family type distribution

**Fig 2:**
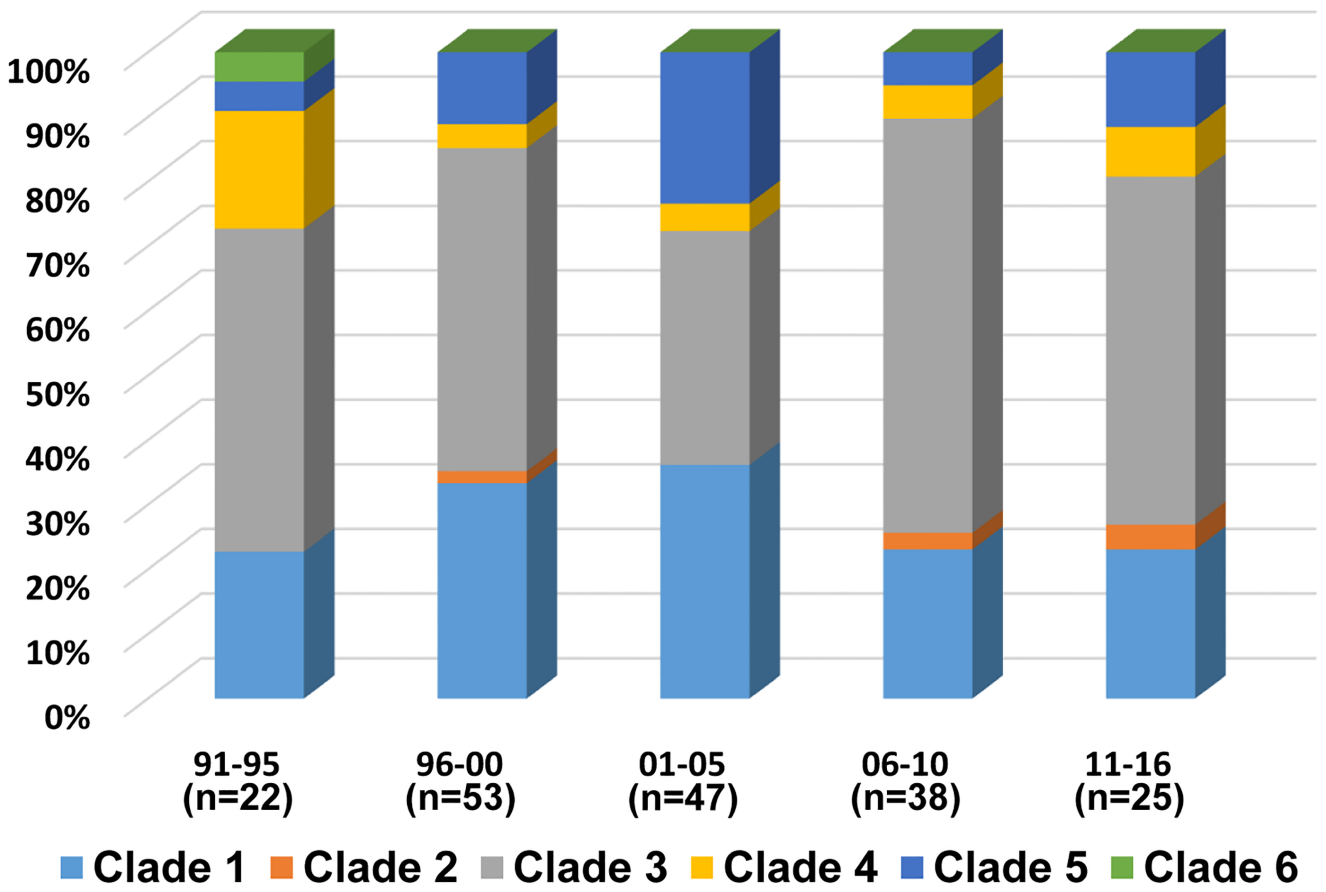
PspA clade types according to the study periods. The x-axis indicates the study periods in five-year intervals (six years in the last period), and the y-axis indicates the cumulative proportion of PspA clade types. The numbers of pneumococcal isolates are presented in parenthesis below the corresponding study period. The color codes for clades 1–6 are shown in the bottom of the figure.

Most PspAs belonged to clade 3 (n = 92, 49.7%) or clade 1 (n = 55, 29.7%). Clades 2, 4, 5, and 6 were identified only in 2 (1.1%), 12 (6.5%), 23 (12.4%), and 1 (0.5%) isolates, respectively. Overall, family 1 (clades 1 and 2), family 2 (clades 3, 4, and 5), and family 3 (clade 6) included 30.8%, 68.7%, and 0.5% of isolates, respectively. The proportion of clade types did not change significantly across the study periods (*P*>0.05 in all; Fig 2). Clade 3 was the most common in all study periods, but the prevalence of clade 1 (36.2%) was the same as that of clade 3 (36.2%) from 2001–2005. Overall, family 2 was dominant (63.8–76.0%) in the invasive pneumococcal isolates obtained from Korean children.

Moreover, the proportion and prevalence of the PspA clades did not change significantly before and after the implementation of PCV7 (March 2003) and PCV10/13 (October 2010) in Korea. In the pre-PCV7 (1991–2003), post-PCV7 (2004–2010), and post-PCV10/13 (2011–2016) eras, the proportions of clade 3 were 43.6%, 57.6%, and 56.0%, respectively (*P*>0.05), and the proportions of clade 1 were 31.7%, 28.8%, and 24.0%, respectively (*P*>0.05).

### Serotype and PspA clade type

**Fig 3:**
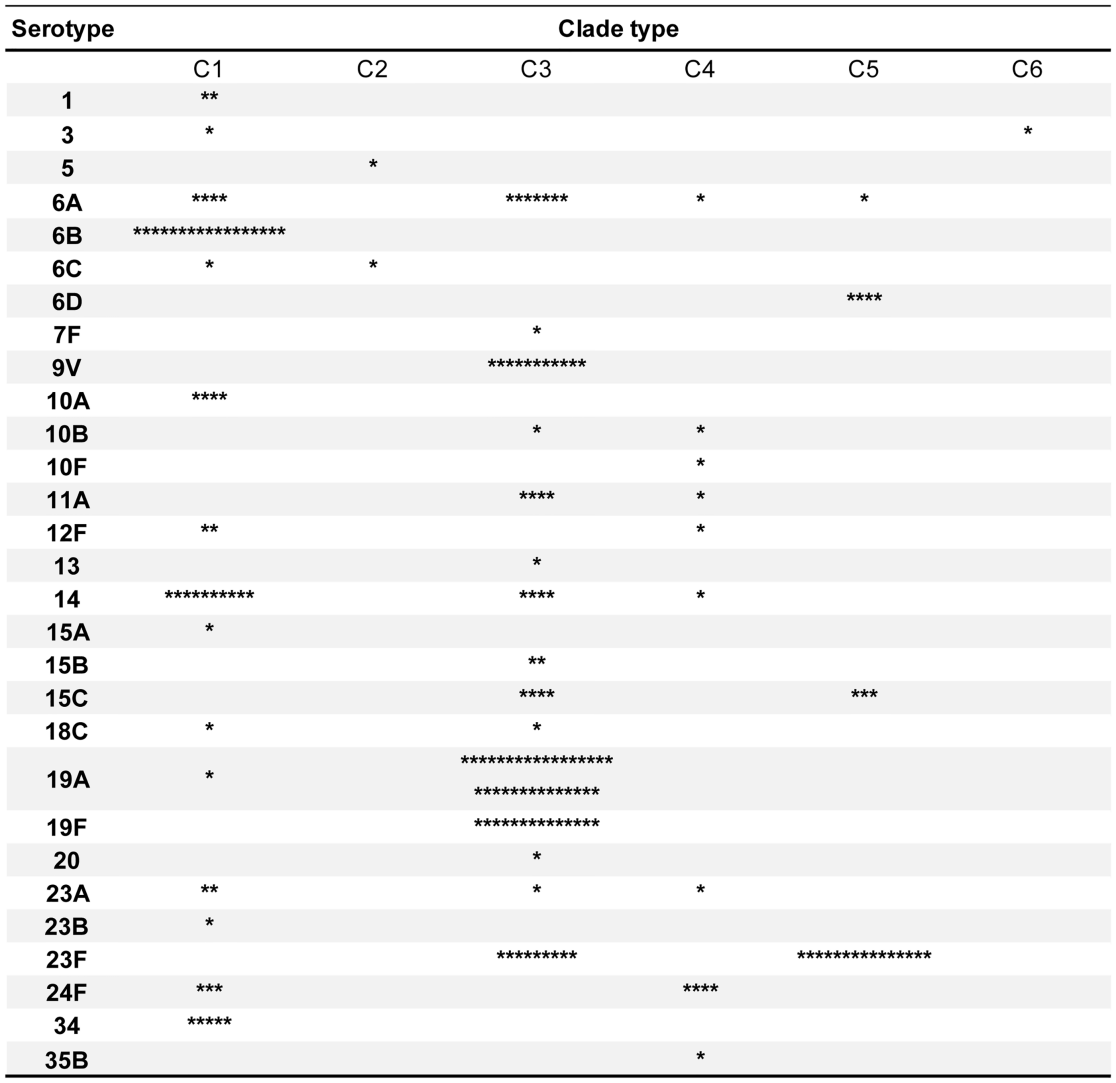
Clade distribution by serotype. Each pneumococcal isolate is indicated by an asterisk (*).

Most serotypes expressed genes from only one or two PspA clades. However, serotypes 14 and 23A expressed genes from three (clades 1, 3, and 4 in both serotypes) and serotype 6A expressed genes from four clades (1, 3, 4, and 5) (Fig 3). The capsular types with most diverse clades (23A [n = 4], 12F [n = 3], and 6A [n = 13]; Simpson’s diversity index [D] = 0.83, 0.67, and 0.65, respectively) were not necessarily those with a large number of isolates (19A [n = 32], 23F [n = 24], and 6B [n = 17]; D = 0.06, 0.50, and 0.00, respectively). The pneumococcal isolates were distributed over all PspA clades, but isolates in serotypes 6B, 6D, 9V, 10A, 19A, 19F, and 34 segregated into particular PspA clade types: clades 1, 5, 3, 1, 3, 3, and 1, respectively.

Regarding the serotype composition of the clades, serotypes 6B (n = 17, 30.9%) and 14 (n = 10, 18.2%) predominated in clade 1; only one isolate of serotypes 5 and 6C was identified in clade 2; serotypes 19A (n = 31, 33.7%), 19F (n = 14, 15.2%), 9V (n = 11, 12.0%), and 23F (n = 9, 9.8%) predominated in clade 3; serotypes 6A, 10B/F, 11A, 12F, 14, 23A, 24F, and 35B prevailed in clade 4; serotype 23F (n = 15, 65.2%) predominated in clade 5; and only one isolate of serotype 3 was identified in clade 6.

### MLST and PspA clade type

**Fig 4:**
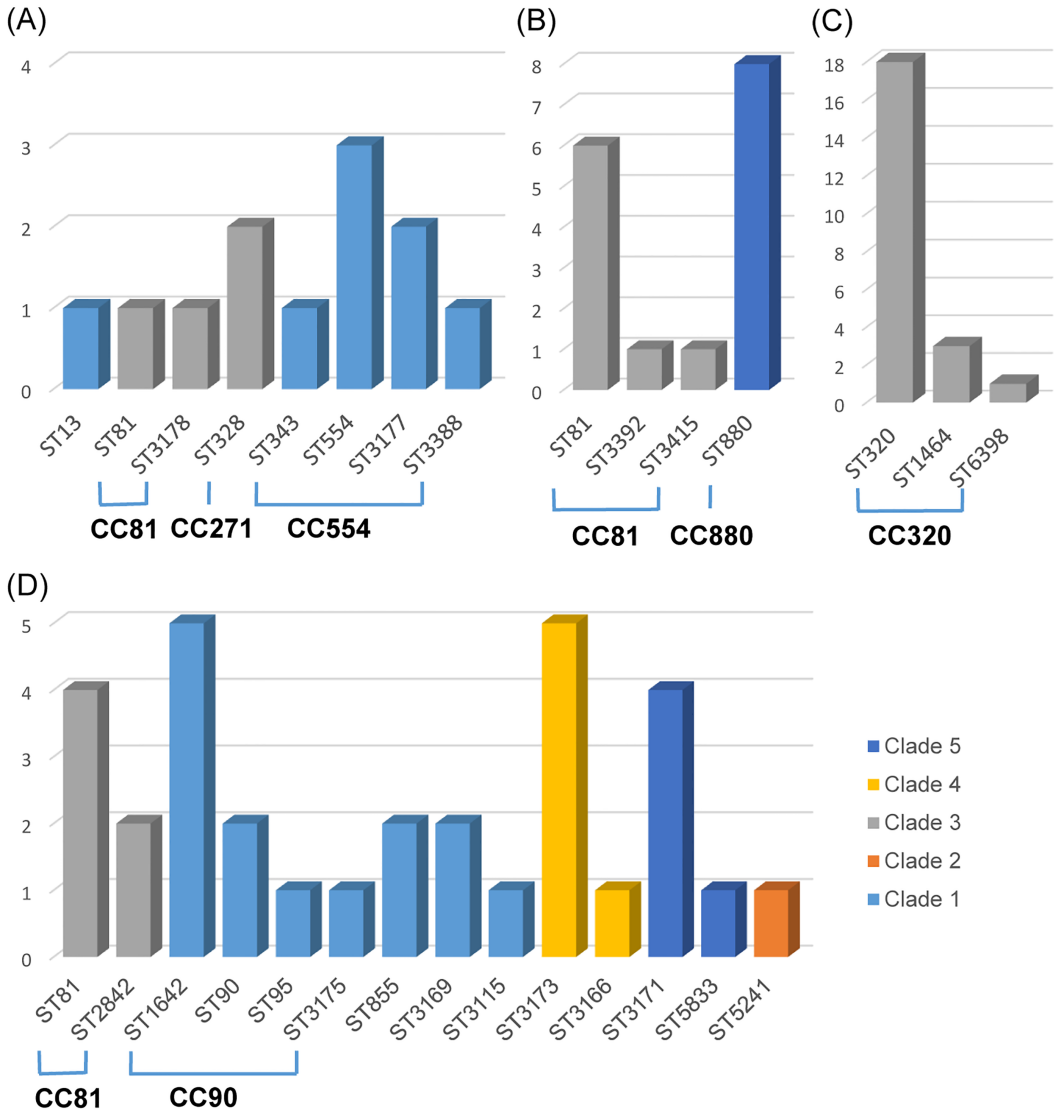
Multilocus sequence type and PspA clade for the serogroup 6 pneumococci. The number of serogroup 6 pneumococcal isolates (y-axis) and their corresponding sequence type (ST, x-axis) is presented with the PspA clade types (differentiated in color). Isolates in the same clonal complex (CC81 or CC90) are exclusively assigned to the same clade type, and each ST including singletons is composed of only one corresponding clade type.

We used an MLST dataset of 120 (64.9%) invasive pneumococcal isolates for comparison with their PspA clade in each serotype. Among the 12 serotypes in the 120 isolates, five serotypes (5, 13, 18C, 34, and 35B) with only one isolate were excluded prior to further analysis. Isolates in serotypes 24F (n = 2, CC90 [n = 1] and ST3393 [n = 1]), 9V (n = 3, all CC166), and 15C (n = 4, all CC81) were assigned to single PspA clades 1, 3, and 3, respectively. Serotype 14 (n = 12) comprised ST13 (n = 1), CC81 (n = 2), CC271 (n = 2), and CC554 (n = 7). The isolates in ST13 and CC554 were all assigned to PspA clade 1, and the isolates in CC81 and CC271 were all assigned to clade 3 (Fig 4(A)). In serotype 23F (n = 16), four sequence types (STs; ST81, ST880, ST3392, and ST3415) and two CCs (CC81 and CC880) were included. Isolates in CC81 (ST81, ST3392, and ST3415; n = 8) and CC880 (n = 8) were assigned to PspA clades 3 and 5, respectively (Fig 4(B)). Serogroup 19 (n = 23; serotypes 19A [n = 19] and 19F [n = 4]) was composed of ST320 (n = 19, 82.6%), ST1464 (n = 3, 13.0%), and ST6398 (n = 1, 4.3%), all of which were included in CC320. All serogroup 19 isolates were exclusively assigned to PspA clade 3 (Fig 4(C)). Serogroup 6 (n = 34) was composed of four serotypes (6A [n = 12], 6B [n = 16], 6C [n = 2], and 6D [n = 4]), 14 STs, and 5 PspA clade types. All serogroup 6 isolates in the ST of singletons were assigned to only one PspA clade. CC81 (ST81 and ST2842; n = 5) and CC90 (ST90, ST95, ST1624 and ST3175; n = 9) of serogroup 6 were exclusively assigned to PspA clades 3 and 1, respectively (Fig 4(D)). In particular, the isolates in CC81 with the different serotypes 15C, 14 (Fig 4(A)), 23F (Fig 4(B)), and 6 (Fig 4(D)) were all assigned to clade 3 of PspA.

### Diversity of the N-terminal α-helical domain

**Fig 5:**
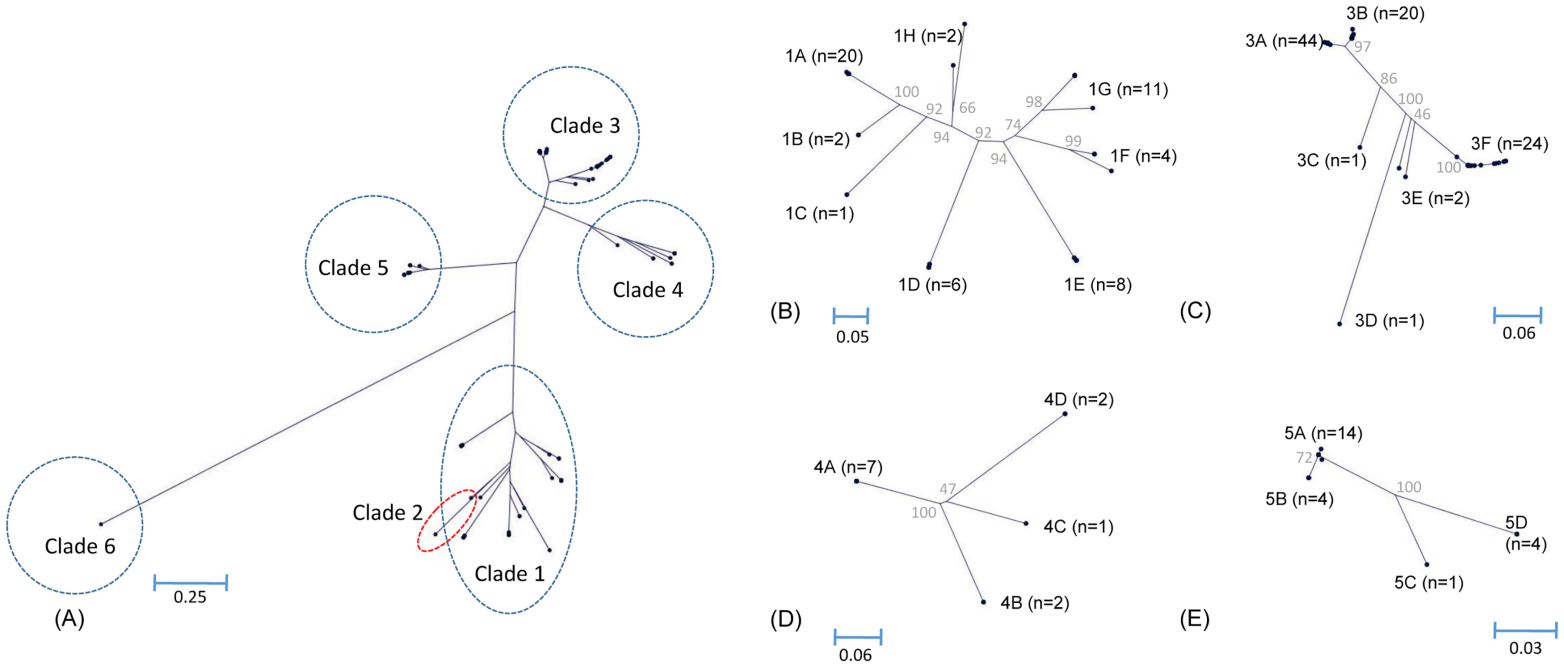
Phylogenetic tree of the N-terminal α-helical domain of PspA. A whole tree (A) with all PspA alleles and extended trees of clades 1 (B), 3 (C), 4 (D), and 5 (E) are shown. Subclades are defined by their common branch of the tree and were assigned alphabetically. Trees of clades 2 (n = 2) and 6 (n = 1) are not presented here because the dendrograms are simple or not able to be constructed, respectively. The bar at the bottom represents genetic distance, and the gray-colored numbers on the tree indicate the bootstrap values (%) on the internal nodes.

The N-terminal α-helical domains were extracted from all 185 PspA sequences. The trees of all of the strains and of subsets of the strains corresponding to clade types, except for clades 2 and 6, which include two and one strains, respectively, were reconstructed (Fig 5). In the complete dendrogram, clade 2/family 1 was close to clade 3/family 2 instead of clade 1/family 1 (Fig 5(A)). Through diversity analysis of the whole α-helical domains, PspA clades 1, 2, 3, 4, and 5 were classified into eight (1A-1H, Fig 5(B)), two (2A and 2B), six (3A-3F, Fig 5(C)), four (4A-4D, Fig 5(D)), and four (5A-5D, Fig 5(E)) subclades, respectively. The genetic diversity of clade 6 was not evaluated as it included only one isolate. PspA subclade 3A was the most prevalent at 23.8% (n = 44) and was present in all study periods, with 2, 8, 10, 15, and 9 isolates in 1991–1995 1996–2000, 2001–2005, 2006–2010, and 2011–2016, respectively. The assignment of the subclades to the isolates is shown in S1 Table.

Pairwise comparisons of nucleotide and amino acid sequences for all 185 PspA α-helical domains showed sequence identities of 45.7–100% and 23.1–100%, respectively. The minimum genetic identities within clades 1, 2, 3, 4, and 5 were 62.2%, 82.2%, 58.7%, 82.0%, and 90.0%, respectively, in nucleotide sequences and 57.8%, 74.0%, 52.2%, 73.8%, and 87.7%, respectively, in amino acid sequences. There were several sequences with maximum genetic identities of 100% in all clades except clade 2.

### Putative antigenicity plots of the N-terminal α-helical domain

**Fig 6:**
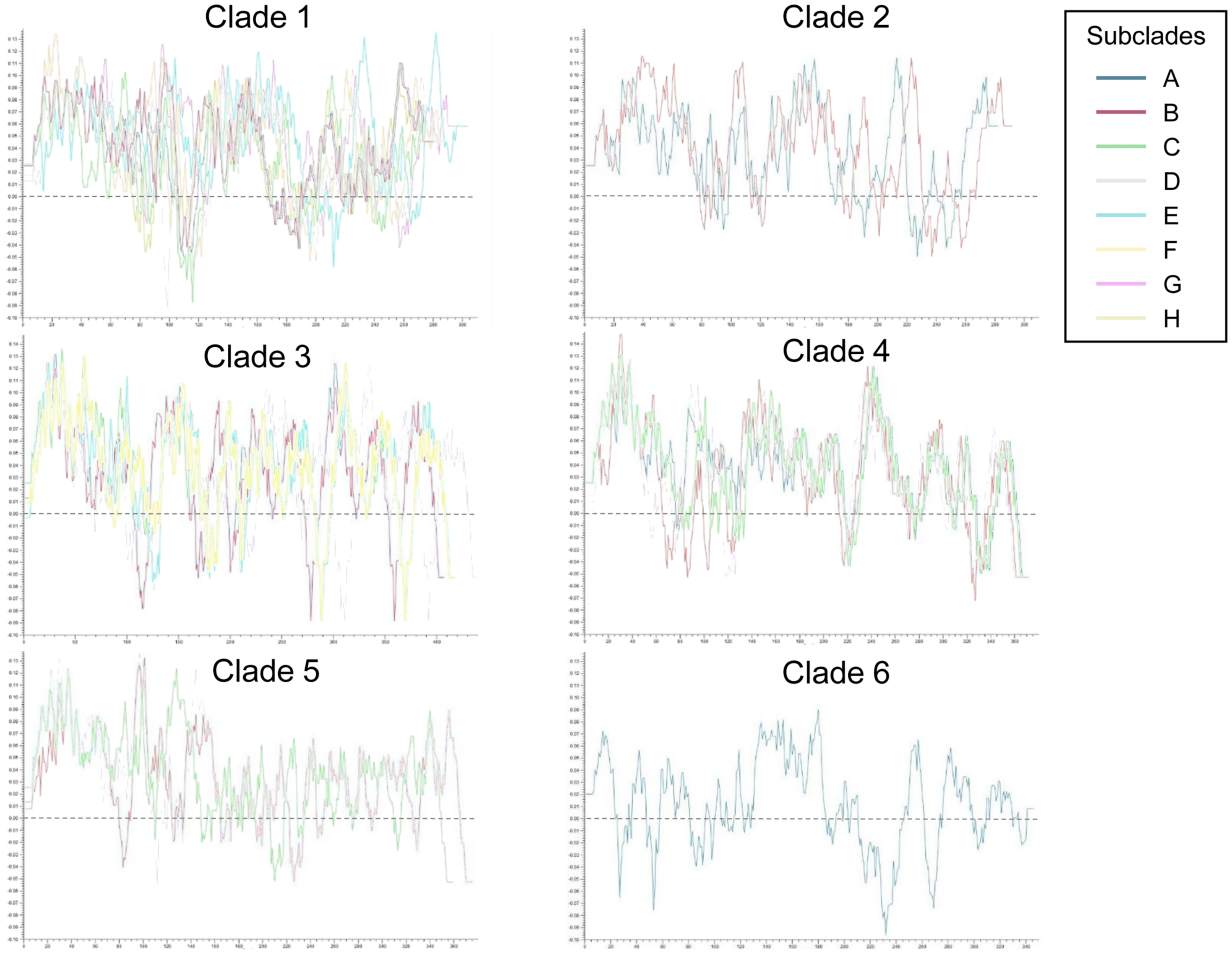
Antigenicity plots of the N-terminal α-helical domain of PspA. The plots for clade types 1–6 are separately presented, and those for subclades (A-H, colored lines) are superimposed in the frame of their corresponding clade type. The x-axis indicates the amino acid position, and the y-axis indicates the hydrophobicity (antigenicity) value. The black dotted horizontal line indicates the neutral point of hydrophobicity.

Representative plots of the individual PspA subclades in different colors were overlapped within their corresponding clade (Fig 6). Clade 1 represented the most diverse antigenicity pattern, and subclades 1E (blue) showed most distinct plot. Although other clades also showed several different positions of peaks due to differences in sequence size, the magnitude and number of major peaks were similar within each clade. In clades 2–5, the differences in antigenicity were most prominent within the N-terminal 120 amino acids. The only isolate of clade 6 showed a unique antigenicity plot. Plots of clades 1, 2, and 5 were positioned higher above the neutral line and showed less negative peaks than those of clades 3, 4, and 6.

## Discussion

In this study, a total of 185 full PspA sequences were acquired from invasive pneumococcal isolates from children. PspA clade 3 and family 2 have been prevalent for 26 years in Korea. Using various molecular biology analyses, we identified that the N-terminal α-helical domain, which is located on the surface of the bacteria and is the major immunogenic portion of PspA, is highly genetically variable among clade types.

The development of a serotype-independent vaccine has been encouraged, and interest has shifted toward surface protein components as vaccine candidates. During the past decade, the immunogenicity and protective capacity of several pneumococcal proteins has been reviewed, and several candidates have been thoroughly investigated, while a few have proceeded to phase I and II clinical trials [18]. PspA has been shown to be highly immunogenic and is a potential candidate for a pneumococcal vaccine. A vaccine composed of PspA is hypothesized to protect against invasive disease and to eliminate the carriage state [8,19,20].

A full exploration of the genetic diversity and molecular epidemiology of PspA in the community is important for its successful implementation as a vaccine candidate. Based on our inferred protein sequences of PspA from 185 strains, 68.7% were PspA family 2, and 49.7% were clade 3/family 2. These findings are in discordance with those of most previous reports on invasive pneumococcal strains, in which PspA family 1 and 2 were detected at similar frequencies; however, these strains were primarily from adult populations [21,22]. Additionally, in studies of children, the two PspA families were similarly detected or family 1 predominated [11,12,23]. However, previous Korean data showed PspA family 1 in 22.5% and family 2 in 66.4% of 89 pneumococcal isolates from children between 1996 and 1998 [24]. In the current study, PspA family 2 was the predominant family during all study periods. In addition, PspA family 2 was found in 71.0% of 69 pneumococcal isolates collected from healthy Malaysian children in 2010 compared to PspA family 1, which was found in 24.6% of the isolates [10]. Thus, it is possible that Asian pneumococcal strains have a predominance of PspA family 2.

PspA clade type was shown to be correlated to the genotype rather than the serotype of the pneumococci strains [11,25]. Although certain PspA types could be dominant in a specific serotype, this correlation might be due to the genotype within the serotype [24,26]. In this study, most serotypes expressed one or two PspA clades, and the isolates of some serotypes were segregated in a particular clade. However, upon comparison of PspA types in several serotypes including their MLST data, we confirmed that the PspA clade type was correlated with the genotype and not the serotype. In addition, the proportion and prevalence of PspA clade types did not change significantly after the use of PCVs in this study, though the prevalence of vaccine serotypes in invasive pneumococcal infections decreased [27,28], which could be a reason for developing a universal vaccine with a pneumococcal protein such as PspA.

PspA is known to be genetically variable between the different family types [7,9]. To establish the possible use of PspA as a vaccine candidate antigen, it is crucial to know the total array and full span of PspAs expressed in pneumococcal strains. We found that 188 (98.9%) of 190 isolates of *S*. *pneumoniae* carried a *pspA* gene whose size ranged from 1,719 to 2,301 bps. The entire span of the *pspA* genes showed 55.7–100% nucleotide identity and 31.3–100% amino acid identity. Although intra-clade sequence identities were higher than the overall identity, they were also more diverse than other pneumococcal protein vaccine candidates such as Ply and PhtD [6].

As previously mentioned, PspA is composed of five domains [7]. Among them, the N-terminal α-helical domain is exposed on the surface, and protection-eliciting epitopes of PspA appear to be spread throughout this region [29]. The induction of antibody production against conformational epitopes present at this region may be important for the promotion of broad protection against pneumococci [30]. A previous study of 40 pneumococcal meningitis isolates from German children showed that the amino acid sequence identity of the α-helical and proline-rich domains were as low as 32% [12]. In the current study, the amino acid sequences in the α-helical domain were much more diverse, and the minimum sequence identity in all pneumococcal isolates was 23.1%.

The grouping of PspA by dendrogram of α-helical domains was different from conventional clade typing based on clade-defining regions. Moreover, the putative antigenicity plots from the α-helical domains were also variable among the PspA clades and subclades. Our previous study showed that Ply and PhtD had nearly identical and very similar putative antigenicity patterns between allele types [6]. However, PspA showed many different positions and peaks on the plot, even in the same clade type, especially in clades 1, 2, and 5. These intra-clade differences in plots were usually located within the N-terminal 120 amino acids. In previous studies, actual immunogenic epitopes of PspA were mapped to regions covering the first 100–115 amino acids [30–32]. In addition, the α-helical domains of clades 2, 4, and 5 showed higher plots than the other clades, so they may be more hydrophobic and immunogenic. Thus, whether the major epitopes of PspA, especially those in the first 120 amino acids of the N-terminal α-helical domain, are conserved and cross-reactive among the various pneumococcal isolates, despite the sequence and antigenic diversities, remains to be confirmed.

In previous studies, the choline-binding domain of PspA was usually not sequenced, as it is known to be relatively invariant [7,12]. However, the entire span of *pspA* was sequenced in this study so that we could explore the diversity of the choline-binding domain. As a result, we found that the 20-amino-acid block in the choline-binding domain was variably repeated 4–15 times. Previous work indicated that the choline-binding domain had 10 repeats of 20 amino acids [7,11], but a strain with 9 repeats in the domain was reviously reported [29]. In this study, which used a large number of pneumococcal strains, we identified that 10 repeats were the most common, followed by 9 and 11 repeats.

In this study, we explored the genetic and antigenic identities of PspA by analyzing its full sequence, specifically focusing on the N-terminal α-helical domain, from 185 pneumococcal isolates. The PspA clade type was correlated with genotype using MLST and was further divided into several subclades. PspA showed variable sequences and antigenic patterns, especially in the immunogenic α-helical domain. As sequence conservation and antigenic epitope stability are necessary requirements for a universal vaccine candidate, further evaluation of the antigenic differences and cross-reactivities between various PspA types is needed, with a particular focus on the genetic diversity of the N-terminal region of PspA.

## Supporting information

S1 Table Strain characteristics including collection period and serotype, PspA size and type, and MLST. (DOCX)
