# Correction: Genetic diversity of pneumococcal surface protein A in invasive pneumococcal isolates from Korean children, 1991-2016

**DOI:** 10.1371/journal.pone.0209511

**Published:** 2018-12-18

**Authors:** Ki Wook Yun, Eun Hwa Choi, Hoan Jong Lee

There are a number of errors in the caption for Fig 4, “Multilocus sequence type and PspA clade for the serogroup 6 pneumococci.” Please see the complete, correct Fig 4 caption here.

**Fig 4:**
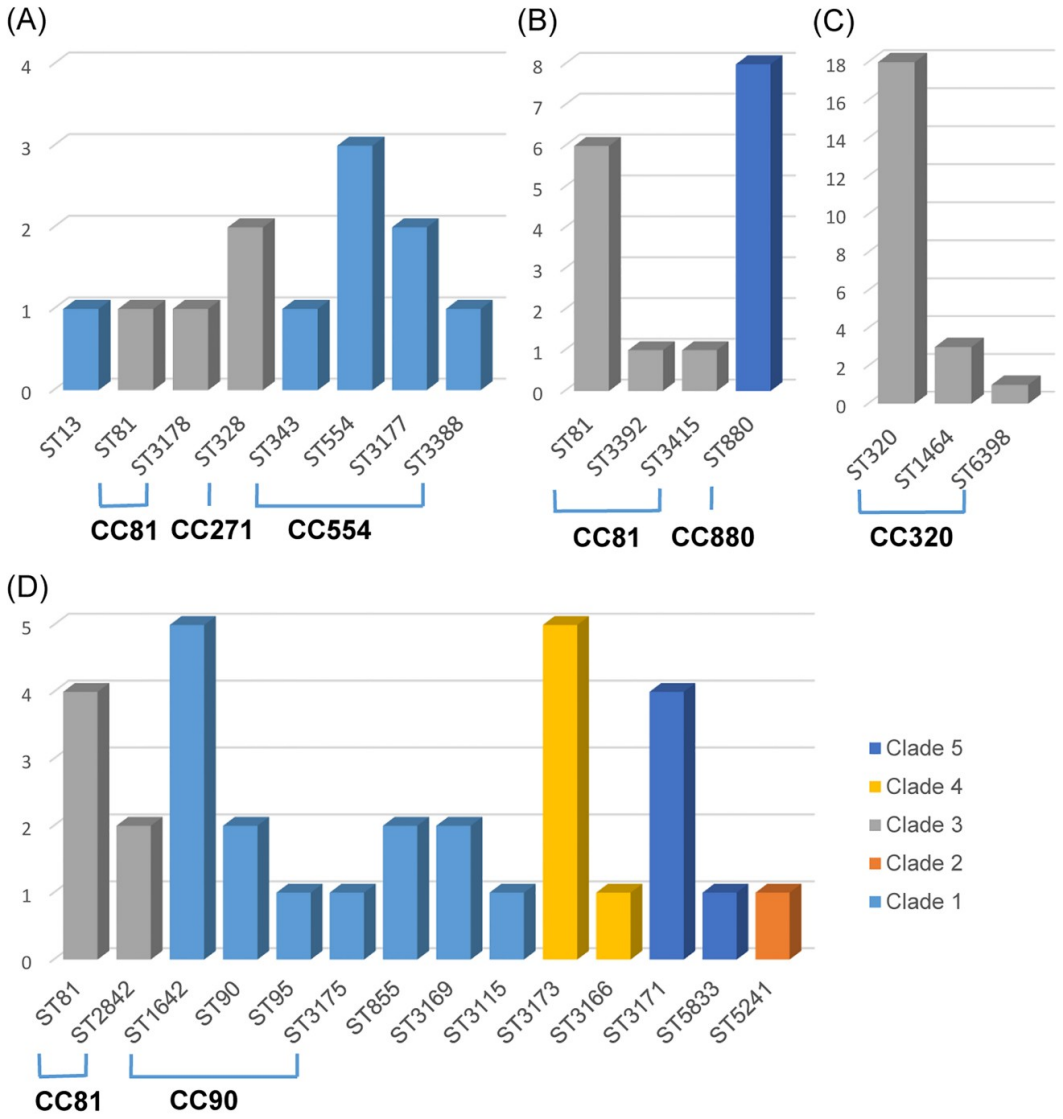
Multilocus sequence type and PspA clade in serogroups 14 (A), 23 (B), 19 (C), and 6 (D) pneumococci. The number pneumococcal isolates (y-axis) in the corresponding sequence type (ST, x-axis) is presented with the PspA clade types (differentiated in color). Isolates in the same clonal complex are exclusively assigned to the same clade type and each ST including singletons is composed of only one corresponding clade type.

## References

[1] Yun KW, Choi EH, Lee HJ (2017) Genetic diversity of pneumococcal surface protein A in invasive pneumococcal isolates from Korean children, 1991–2016. PLoS ONE 12(11): e0183968 10.1371/journal.pone.0183968 29131872 PMC5683564

