# Supplementary material for: Genetic diversity of pneumococcal surface protein A in invasive pneumococcal isolates from Korean children, 1991-2016
Source: PLoS One. 2017 Nov 13;12(11):e0183968. doi: 10.1371/journal.pone.0183968 (PMC5683564; doi:10.1371/journal.pone.0183968)
Supplement: S1 Table — (DOCX) [file pone.0183968.s001.docx]

Supplement Table S1. Strain characteristics including collection period and serotype, PspA size and type, and MLST

| **Strain** | **Period (year)** | **Serotype** | **PCV type** | **PspA** | | | | **MLST** | |
| --- | --- | --- | --- | --- | --- | --- | --- | --- | --- |
|  |  |  |  | **Size (bp)** | **Family** | **Clade** | **Subtype** | **CC** | **ST** |
| 91-022 | 91-95 | 24F | NVT | 2094 | 2 | 4 | 4A |  |  |
| 91-026 | 91-95 | 23F | PCV7 | 2166 | 2 | 5 | 5B |  |  |
| 92-099 | 91-95 | 24F | NVT | 1899 | 1 | 1 | 1F |  |  |
| 92-153 | 91-95 | 15C | NVT | 2262 | 2 | 3 | 3B |  |  |
| 93-009 | 91-95 | 19F | PCV7 | 2175 | 2 | 3 | 3A |  |  |
| 93-012 | 91-95 | 15B | NVT | 2019 | 2 | 3 | 3B |  |  |
| 93-036 | 91-95 | 24F | NVT | 2094 | 2 | 4 | 4A |  |  |
| 94-004 | 91-95 | 3 | PCV13 | 2019 | 1 | 1 | 1F |  |  |
| 94-022 | 91-95 | 18C | PCV7 | 1848 | 1 | 1 | 1H |  |  |
| 94-072 | 91-95 | 9V | PCV7 | 2214 | 2 | 3 | 3B |  |  |
| 94-079 | 91-95 | 10B | NVT | 2241 | 2 | 3 | 3E |  |  |
| 94-089 | 91-95 | 3 | PCV13 | 2169 | 3 | 6 | 6A |  |  |
| 94-110 | 91-95 | 10B | NVT | 2109 | 2 | 4 | 4B |  |  |
| 94-115 | 91-95 | 9V | PCV7 | 2241 | 2 | 3 | 3B |  |  |
| 95-035 | 91-95 | 19F | PCV7 | 2241 | 2 | 3 | 3B |  |  |
| 95-039 | 91-95 | 14 | PCV7 | 1935 | 2 | 3 | 3F | 81 | 3178 |
| 95-072 | 91-95 | 6A | PCV13 | 1839 | 1 | 1 | 1A | 3115 | 3115 |
| 95-077 | 91-95 | 19F | PCV7 | 2175 | 2 | 3 | 3A |  |  |
| 95-084 | 91-95 | 23F | PCV7 | 2178 | 2 | 3 | 3F | 81 | 81 |
| 95-124 | 91-95 | 6A | PCV13 | 2124 | 2 | 4 | 4D | S | 3166 |
| 95-140 | 91-95 | 9V | PCV7 | 2187 | 2 | 3 | 3B |  |  |
| 95-141 | 91-95 | 6A | PCV13 | 1839 | 1 | 1 | 1A | 3115 | 3115 |
| 96-012 | 96-00 | 23F | PCV7 | 2178 | 2 | 3 | 3F | 81 | 3415 |
| 96-038 | 96-00 | 19F | PCV7 | 2175 | 2 | 3 | 3A | 271 | 271 |
| 96-039 | 96-00 | 14 | PCV7 | 1935 | 2 | 3 | 3F | 81 | 81 |
| 96-043 | 96-00 | 10F | NVT | 2109 | 2 | 4 | 4B |  |  |
| 96-047 | 96-00 | 6D | NVT | 2049 | 2 | 5 | 5D | S | 3171 |
| 96-048 | 96-00 | 1 | PCV13 | 1836 | 1 | 1 | 1B |  |  |
| 96-049 | 96-00 | 14 | PCV7 | 1869 | 1 | 1 | 1G | 554 | 343 |
| 96-050 | 96-00 | 14 | PCV7 | 1869 | 1 | 1 | 1G |  |  |
| 96-067 | 96-00 | 24F | NVT | 1839 | 1 | 1 | 1F | S | 3393 |
| 96-091 | 96-00 | 34 | NVT | 1821 | 1 | 1 | 1D |  |  |
| 96-095 | 96-00 | 14 | PCV7 | 2175 | 2 | 3 | 3A | 271 | 328 |
| 96-098 | 96-00 | 23F | PCV7 | 1935 | 2 | 3 | 3F | 81 | 81 |
| 97-002 | 96-00 | 10A | NVT | 1851 | 1 | 1 | 1C |  |  |
| 97-010 | 96-00 | 19F | PCV7 | 2175 | 2 | 3 | 3A | S | 2399 |
| 97-011 | 96-00 | 14 | PCV7 | 2175 | 2 | 3 | 3A | 271 | 328 |
| 97-019 | 96-00 | 6B | PCV7 | 1887 | 1 | 1 | 1D | S | 3169 |
| 97-033 | 96-00 | 23F | PCV7 | 2178 | 2 | 3 | 3F | 81 | 81 |
| 97-041 | 96-00 | 6D | NVT | 2049 | 2 | 5 | 5D | S | 3171 |
| 97-054 | 96-00 | 6D | NVT | 2049 | 2 | 5 | 5D | S | 3171 |
| 97-055 | 96-00 | 23F | PCV7 | 2178 | 2 | 3 | 3F | 81 | 81 |
| 97-056 | 96-00 | 19F | PCV7 | 2247 | 2 | 3 | 3D | 271 | 236 |
| 97-057 | 96-00 | 6D | NVT | 2049 | 2 | 5 | 5D | S | 3171 |
| 97-064 | 96-00 | 6B | PCV7 | 1899 | 1 | 1 | 1A | 90 | 90 |
| 97-092 | 96-00 | 6B | PCV7 | 1899 | 1 | 1 | 1A | 90 | 1624 |
| 97-097 | 96-00 | 19A | PCV13 | 1953 | 1 | 1 | 1A | S | 1374 |
| 97-099 | 96-00 | 12F | NVT | 1911 | 1 | 1 | 1G |  |  |
| 97-102 | 96-00 | 23F | PCV7 | 2166 | 2 | 5 | 5A | 880 | 880 |
| 97-113 | 96-00 | 23F | PCV7 | 2238 | 2 | 3 | 3F | 81 | 3392 |
| 98-001 | 96-00 | 14 | PCV7 | 1869 | 1 | 1 | 1G | 554 | 554 |
| 98-015 | 96-00 | 11A | NVT | 2241 | 2 | 3 | 3B |  |  |
| 98-021 | 96-00 | 23F | PCV7 | 2178 | 2 | 3 | 3F | 81 | 81 |
| 98-038 | 96-00 | 1 | PCV13 | 1836 | 1 | 1 | 1B |  |  |
| 98-090 | 96-00 | 9V | PCV7 | 2241 | 2 | 3 | 3B | 166 | 166 |
| 99-003 | 96-00 | 6B | PCV7 | 1899 | 1 | 1 | 1A | 90 | 90 |
| 99-010 | 96-00 | 9V | PCV7 | 2241 | 2 | 3 | 3B |  |  |
| 99-018 | 96-00 | 23F | PCV7 | 2166 | 2 | 5 | 5A | 880 | 880 |
| 99-036 | 96-00 | 5 | PCV13 | 1926 | 1 | 2 | 2A | S | 3394 |
| 99-049 | 96-00 | 19A | PCV13 | 2175 | 2 | 3 | 3A | 271 | 320 |
| 99-052 | 96-00 | 19F | PCV7 | 2175 | 2 | 3 | 3A |  |  |
| 99-128 | 96-00 | 14 | PCV7 | 1869 | 1 | 1 | 1G | 554 | 554 |
| 00-001 | 96-00 | 9V | PCV7 | 2241 | 2 | 3 | 3B | 166 | 3383 |
| 00-012 | 96-00 | 14 | PCV7 | 1857 | 1 | 1 | 1G | 554 | 3388 |
| 00-022 | 96-00 | 19F | PCV7 | 2175 | 2 | 3 | 3A | 271 | 271 |
| 00-043 | 96-00 | 9V | PCV7 | 2301 | 2 | 3 | 3B | 166 | 166 |
| 00-050 | 96-00 | 14 | PCV7 | 1869 | 1 | 1 | 1G |  |  |
| 00-058 | 96-00 | 6A | PCV13 | 2178 | 2 | 3 | 3F | 81 | 81 |
| 00-088 | 96-00 | 6A | PCV13 | 2178 | 2 | 3 | 3F | 81 | 81 |
| 00-093 | 96-00 | 19A | PCV13 | 2175 | 2 | 3 | 3A | 271 | 320 |
| 00-100 | 96-00 | 23F | PCV7 | 2052 | 2 | 3 | 3F | 81 | 81 |
| 00-109 | 96-00 | 15C | NVT | 2178 | 2 | 3 | 3F | 81 | 83 |
| 00-143 | 96-00 | 35B | NVT | 2130 | 2 | 4 | 4D | S | 558 |
| 00-149 | 96-00 | 6B | PCV7 | 1896 | 1 | 1 | 1E | S | 3173 |
| 00-186 | 96-00 | 15C | NVT | 1935 | 2 | 3 | 3F | 81 | 83 |
| 01-010 | 01-05 | 6B | PCV7 | 1887 | 1 | 1 | 1D | S | 3169 |
| 01-017 | 01-05 | 6B | PCV7 | 1899 | 1 | 1 | 1A | 90 | 95 |
| 01-024 | 01-05 | 19A | PCV13 | 2175 | 2 | 3 | 3A | 271 | 320 |
| 01-053 | 01-05 | 6B | PCV7 | 1878 | 1 | 1 | 1E | S | 3173 |
| 01-062 | 01-05 | 24F | NVT | 2094 | 2 | 4 | 4A |  |  |
| 01-063 | 01-05 | 23F | PCV7 | 2166 | 2 | 5 | 5A | 880 | 880 |
| 01-092 | 01-05 | 19A | PCV13 | 2175 | 2 | 3 | 3A | 271 | 320 |
| 01-161 | 01-05 | 23F | PCV7 | 2166 | 2 | 5 | 5A |  |  |
| 01-189 | 01-05 | 6C | NVT | 2157 | 1 | 1 | 1G | S | 3168 |
| 01-201 | 01-05 | 19F | PCV7 | 2175 | 2 | 3 | 3A | 271 | 271 |
| 01-214 | 01-05 | 23F | PCV7 | 2166 | 2 | 5 | 5A | 880 | 880 |
| 01-264 | 01-05 | 23F | PCV7 | 2166 | 2 | 5 | 5A |  |  |
| 01-274 | 01-05 | 23F | PCV7 | 2166 | 2 | 5 | 5A |  |  |
| 01-325 | 01-05 | 19A | PCV13 | 2175 | 2 | 3 | 3A | 271 | 320 |
| 02-075 | 01-05 | 23F | PCV7 | 2166 | 2 | 5 | 5A |  |  |
| 02-076 | 01-05 | 34 | NVT | 1806 | 1 | 1 | 1D | S | 3116 |
| 02-240 | 01-05 | 14 | PCV7 | 1869 | 1 | 1 | 1G | 554 | 554 |
| 02-281 | 01-05 | 19A | PCV13 | 2175 | 2 | 3 | 3A | 271 | 320 |
| 02-289 | 01-05 | 34 | NVT | 1887 | 1 | 1 | 1D |  |  |
| 02-467 | 01-05 | 6B | PCV7 | 1860 | 1 | 1 | 1E | S | 3173 |
| 02-519 | 01-05 | 19F | PCV7 | 2175 | 2 | 3 | 3A | 271 | 236 |
| 02-523 | 01-05 | 19A | PCV13 | 2175 | 2 | 3 | 3A | 271 | 320 |
| 02-556 | 01-05 | 24F | NVT | 1899 | 1 | 1 | 1A | 90 | 3387 |
| 02-593 | 01-05 | 6A | PCV13 | 2118 | 2 | 5 | 5C | S | 5833 |
| 02-624 | 01-05 | 23F | PCV7 | 2166 | 2 | 5 | 5A | 880 | 880 |
| 02-700 | 01-05 | 23F | PCV7 | 2166 | 2 | 5 | 5A | 880 | 880 |
| 03-006 | 01-05 | 23F | PCV7 | 2166 | 2 | 5 | 5A | 880 | 880 |
| 03-020 | 01-05 | 14 | PCV7 | 1908 | 1 | 1 | 1G | 554 | 3177 |
| 03-030 | 01-05 | 19A | PCV13 | 2175 | 2 | 3 | 3A | 271 | 320 |
| 03-043 | 01-05 | 18C | PCV7 | 2178 | 2 | 3 | 3F | S | 3180 |
| 03-072 | 01-05 | 20 | NVT | 2235 | 2 | 3 | 3B |  |  |
| 03-089 | 01-05 | 14 | PCV7 | 1770 | 1 | 1 | 1H | S | 13 |
| 03-126 | 01-05 | 9V | PCV7 | 2268 | 2 | 3 | 3B |  |  |
| 03-152 | 01-05 | 6A | PCV13 | 1779 | 1 | 1 | 1A | S | 855 |
| 03-176 | 01-05 | 19A | PCV13 | 2175 | 2 | 3 | 3A | 271 | 320 |
| 03-245 | 01-05 | 13 | NVT | 2178 | 2 | 3 | 3F | 81 | 189 |
| 03-290 | 01-05 | 6B | PCV7 | 1899 | 1 | 1 | 1A | 90 | 1624 |
| 03-292 | 01-05 | 6A | PCV13 | 2178 | 2 | 3 | 3F | 81 | 2842 |
| 04-073 | 01-05 | 6A | PCV13 | 1779 | 1 | 1 | 1A | S | 855 |
| 04-100 | 01-05 | 19A | PCV13 | 2175 | 2 | 3 | 3A | 554 | 343 |
| 04-154 | 01-05 | 23F | PCV7 | 2166 | 2 | 5 | 5A | 880 | 880 |
| 04-215 | 01-05 | 12F | NVT | 2196 | 2 | 4 | 4A |  |  |
| 05-077 | 01-05 | 14 | PCV7 | 1908 | 1 | 1 | 1G | 554 | 3177 |
| 05-097 | 01-05 | 6B | PCV7 | 1899 | 1 | 1 | 1A | 90 | 3175 |
| 05-175 | 01-05 | 15C | NVT | 1935 | 2 | 3 | 3F | 81 | 83 |
| 05-233 | 01-05 | 6A | PCV13 | 2178 | 2 | 3 | 3F | 81 | 81 |
| 05-276 | 01-05 | 6B | PCV7 | 1899 | 1 | 1 | 1A | 90 | 6917 |
| 06-083 | 06-10 | 14 | PCV7 | 2169 | 2 | 4 | 4A |  |  |
| 06-112 | 06-10 | 6B | PCV7 | 1896 | 1 | 1 | 1E | S | 3173 |
| 06-113 | 06-10 | 23A | NVT | 1719 | 1 | 1 | 1A |  |  |
| 06-151 | 06-10 | 23A | NVT | 1839 | 1 | 1 | 1A |  |  |
| 06-224 | 06-10 | 19A | PCV13 | 2175 | 2 | 3 | 3A |  |  |
| 06-237 | 06-10 | 19A | PCV13 | 2148 | 2 | 3 | 3A |  |  |
| 06-245 | 06-10 | 19F | PCV7 | 2175 | 2 | 3 | 3A | 271 | 1464 |
| 07-001 | 06-10 | 19F | PCV7 | 2175 | 2 | 3 | 3A | 271 | 320 |
| 07-030 | 06-10 | 12F | NVT | 1896 | 1 | 1 | 1E |  |  |
| 07-059 | 06-10 | 6B | PCV7 | 1896 | 1 | 1 | 1E | S | 3173 |
| 07-065 | 06-10 | 19A | PCV13 | 2175 | 2 | 3 | 3A | 271 | 320 |
| 07-070 | 06-10 | 7F | PCV13 | 2358 | 2 | 3 | 3C |  |  |
| 07-092 | 06-10 | 19A | PCV13 | 2175 | 2 | 3 | 3A | 271 | 320 |
| 07-094 | 06-10 | 9V | PCV7 | 2268 | 2 | 3 | 3B |  |  |
| 07-097 | 06-10 | 6B | PCV7 | 1899 | 1 | 1 | 1A | 90 | 1624 |
| 07-184 | 06-10 | 19A | PCV13 | 2202 | 2 | 3 | 3A | 271 | 320 |
| 08-010 | 06-10 | 9V | PCV7 | 2214 | 2 | 3 | 3B |  |  |
| 08-015 | 06-10 | 23F | PCV7 | 1935 | 2 | 3 | 3F |  |  |
| 08-016 | 06-10 | 19A | PCV13 | 2175 | 2 | 3 | 3A | 271 | 6398 |
| 08-207 | 06-10 | 6C | NVT | 1881 | 1 | 2 | 2B | S | 5241 |
| 08-213 | 06-10 | 15C | NVT | 2166 | 2 | 5 | 5B |  |  |
| 08-443 | 06-10 | 6B | PCV7 | 1899 | 1 | 1 | 1A | 90 | 1624 |
| 08-445 | 06-10 | 9V | PCV7 | 2268 | 2 | 3 | 3B |  |  |
| 09-163 | 06-10 | 19F | PCV7 | 2148 | 2 | 3 | 3A | 271 | 1464 |
| 09-194 | 06-10 | 19A | PCV13 | 2127 | 2 | 3 | 3E | 271 | 320 |
| 09-218 | 06-10 | 19A | PCV13 | 2175 | 2 | 3 | 3A | 271 | 320 |
| 09-437 | 06-10 | 11A | NVT | 2091 | 2 | 4 | 4C |  |  |
| 10-112 | 06-10 | 6A | PCV13 | 2178 | 2 | 3 | 3F | 81 | 81 |
| 10-149 | 06-10 | 10A | NVT | 1899 | 1 | 1 | 1A |  |  |
| 10-166 | 06-10 | 11A | NVT | 2241 | 2 | 3 | 3B |  |  |
| 10-200 | 06-10 | 19A | PCV13 | 2175 | 2 | 3 | 3A | 271 | 320 |
| 10-434 | 06-10 | 34 | NVT | 1827 | 1 | 1 | 1D |  |  |
| 10-444 | 06-10 | 15C | NVT | 2166 | 2 | 5 | 5B |  |  |
| 10-478 | 06-10 | 19A | PCV13 | 2175 | 2 | 3 | 3A | 271 | 320 |
| 10-489 | 06-10 | 19A | PCV13 | 2148 | 2 | 3 | 3A | 271 | 320 |
| 10-512 | 06-10 | 6A | PCV13 | 1935 | 2 | 3 | 3F | 81 | 2842 |
| 10-518 | 06-10 | 19A | PCV13 | 2175 | 2 | 3 | 3A | 271 | 320 |
| 10-539 | 06-10 | 19A | PCV13 | 2148 | 2 | 3 | 3A | 271 | 320 |
| 11-092 | 11-16 | 23F | PCV7 | 2166 | 2 | 5 | 5A |  |  |
| 11-127 | 11-16 | 19A | PCV13 | 1905 | 2 | 3 | 3A | 271 | 320 |
| 11-128 | 11-16 | 19A | PCV13 | 1905 | 2 | 3 | 3A | 271 | 320 |
| 11-136 | 11-16 | 19A | PCV13 | 2175 | 2 | 3 | 3A | 271 | 320 |
| 11-162 | 11-16 | 6B | PCV7 | 1779 | 1 | 1 | 1A | 90 | 1624 |
| 11-165 | 11-16 | 19A | PCV13 | 2175 | 2 | 3 | 3A | 271 | 320 |
| 11-183 | 11-16 | 19A | PCV13 | 1785 | 2 | 3 | 3A | 271 | 320 |
| 11-201 | 11-16 | 19A | PCV13 | 2175 | 2 | 3 | 3A | 271 | 320 |
| 12-243 | 11-16 | 19A | PCV13 | 2175 | 2 | 3 | 3A | 271 | 320 |
| 12-248 | 11-16 | 19F | PCV7 | 2175 | 2 | 3 | 3A | 271 | 1464 |
| 13-133 | 11-16 | 19A | PCV13 | 2175 | 2 | 3 | 3A | 271 | 320 |
| 13-143 | 11-16 | 23B | NVT | 1782 | 1 | 1 | 1F |  |  |
| 13-255 | 11-16 | 11 | NVT | 2187 | 2 | 3 | 3B |  |  |
| 14-036 | 11-16 | 23F | PCV7 | 2166 | 2 | 5 | 5A |  |  |
| 14-044 | 11-16 | 24F | NVT | 2094 | 2 | 4 | 4A |  |  |
| 14-095 | 11-16 | 11A | NVT | 2241 | 2 | 3 | 3B |  |  |
| 14-175 | 11-16 | 15C | NVT | 2166 | 2 | 5 | 5B |  |  |
| 15-049 | 11-16 | 6A | PCV13 | 2178 | 2 | 3 | 3F | 81 | 81 |
| 15-328 | 11-16 | 15A | NVT | 1839 | 1 | 1 | 1A |  |  |
| 16-008 | 11-16 | 23A | NVT | 2094 | 2 | 4 | 4A |  |  |
| 16-043 | 11-16 | 34 | NVT | 1827 | 1 | 1 | 1D |  |  |
| 16-086 | 11-16 | 15B | NVT | 2178 | 2 | 3 | 3F |  |  |
| 16-110 | 11-16 | 10* | NVT | 1950 | 1 | 1 | 1E |  |  |
| 16-111 | 11-16 | 10* | NVT | 1950 | 1 | 1 | 1E |  |  |
| 16-201 | 11-16 | 23A | NVT | 2187 | 2 | 3 | 3B |  |  |
